# Feline Adipose Derived Multipotent Stromal Cell Transdifferentiation Into Functional Insulin Producing Cell Clusters

**DOI:** 10.3389/fbioe.2022.904519

**Published:** 2022-06-08

**Authors:** Takashi Taguchi, Wei Duan, Wendy Wolfson, Brandy Duhon, Emily G. Halphen, Mandi J. Lopez

**Affiliations:** ^1^ Laboratory for Equine and Comparative Orthopedic Research, Department of Veterinary Clinical Sciences, School of Veterinary Medicine, Louisiana State University, Baton Rouge, LA, United States; ^2^ Department of Veterinary Clinical Sciences, School of Veterinary Medicine, Louisiana State University, Baton Rouge, LA, United States

**Keywords:** glucose, beta cell (β-cell), stem cell, diabetes, pancreas, endocrine

## Abstract

Diabetes mellitus (DM) is one of the most prevalent feline endocrinopathies, affecting up to 1% of pet cats. De novo generation of functional insulin producing cell (IPC) clusters *via* transdifferentiation of feline adipose-derived multipotent stromal cells (ASCs) may not only provide a viable, functional cell therapy for feline DM, but may also serve as a platform for developing a comparable human treatment given feline and human DM similarities. Cells were induced to form IPCs with a novel, three-stage culture process with stromal or differentiation medium under static and dynamic conditions. Clusters were evaluated for intracellular zinc, viability, intracellular insulin, glucagon, and somatostatin, ultrastructure, glucose stimulated insulin secretion in the presence or absence of theophylline, and protein and gene expression. Isolated cells were multipotent, and cell clusters cultured in both media had robust cell viability. Those cultured in differentiation medium contained zinc and mono- or polyhormonal α-, β-, and δ-like cells based on immunohistochemical labeling and Mallory-Heidenhan Azan-Gomori’s staining. Ultrastructurally, cell clusters cultured in differentiation medium contained insulin granules within vesicles, and clusters had a concentration-dependent insulin response to glucose in the presence and absence of theophylline which increased both insulin secretion and intracellular content. Expression of NK6.1, Pax6, Isl1, Glut2, RAB3A, glucagon, insulin, and somatostatin increased with differentiation stage for both sexes, and expression of nestin at stages 1 and 2 and Neurod1 at stage 2 was higher in cells from female donors. The cluster insulin secretion responses and endocrine and oncogene gene expression profiles were inconsistent with insulinoma characteristics. A total of 180 proteins were upregulated in differentiated clusters, and the majority were associated with biological regulation, metabolic processes, or stimulus response. Dynamic culture of IPC clusters resulted in clusters composed of cells primarily expressing insulin that released higher insulin with glucose stimulation than those in static culture. Collectively, the results of this study support generation of functional IPC clusters using feline ASCs isolated from tissues removed during routine sterilization. Further, cluster functionality is enhanced with dynamic, motion-driven shear stress. This work establishes a foundation for development of strategies for IPC therapy for short or long-term diabetes treatment and may represent an option to study prevention and treatment of diabetes across species.

## Introduction

Diabetes mellitus is one of the most prevalent feline endocrinopathies (Goossens et al., 1998; Rand et al., 2004). To date, there is no single cause or effective cure. Serious complications associated with unregulated glucose levels include bone fractures (Schwartz, 2003), cardiovascular disease (Shehadeh and Regan, 1995), and neurological dysfunction (Mizisin et al., 2002), among others. Contemporary treatment consists of diet and weight management with exogenous insulin administration, including commercially available synthetic formulations, to replace that normally produced by pancreatic *β* cells (Fu et al., 2013). Though insulin maintains biological activity across species, sequence differences may impact both activity and immunogenicity (Chance et al., 1968; Betsholtz et al., 1990; Fineberg et al., 2007). Additionally, insulin administration must be continually customized for individual patients, a challenging and time-consuming process.

One approach to address the limitations of exogenous insulin therapy is pancreatic islet transplantation. Reportedly, 50%–70% of human type I diabetics that received pancreatic islet implants did not require insulin therapy 5 years after treatment (Ryan et al., 2004b; Shapiro et al., 2017). Islet transplantation in dogs with type I diabetes resulted in up to a 50% reduction in insulin dose and improved glycemic control 6 months post-implantation (Gooch et al., 2019). In contrast, allogenic feline islets implanted into pancreatectomized recipients conferred only 12 days of normoglycemia before implant rejection (Maeno et al., 2006). Additional limitations of islet transplants in feline patients are similar to those in other species including limited availability, risk of disease transmission, and the need for recipient immunosuppression (Ryan et al., 2004a). An alternative to allogenic islet implantation is *de novo* generation of pancreatic cells from progenitor cells, largely accomplished thus far with embryonic, and induced pluripotent stem cells (Rezania et al., 2012; Pagliuca et al., 2014). Ethical concerns, potential risks of gene editing, and allogenic immune reactions complicate mainstream implementation of embryonic and induced pluripotent cell-based tissue implants (Doss and Sachinidis, 2019).

Autologous adult multipotent stromal cells (MSCs) may provide another option for *de novo* pancreatic cell generation. Due to the ability to differentiate into multiple tissues (Webb et al., 2012; Kono et al., 2014; Zhang et al., 2014), MSCs are popular for cell therapies designed to restore tissues lost to trauma or disease. Current knowledge also supports the ability of MSCs to transdifferentiate into tissues derived from other embryonic layers (Buang et al., 2012; Dave et al., 2013; Moshtagh et al., 2013). Differentiation of mesodermal adipose tissue-derived multipotent stromal cells (ASCs) into endodermal insulin producing cell (IPC) clusters is a contemporary example of transdifferentiation confirmed in several species (Chen et al., 2004; Dave et al., 2013; Dubey et al., 2014). Further, an established mechanism to isolate a high yield of feline ASCs from adipose tissues removed with reproductive organs during routine feline sterilization creates a unique opportunity to partner routine tissue extraction with a treatment for a ubiquitous and challenging endocrine pathology (Zhang et al., 2014). However, given the endocrine function of adipose tissue, potential differences in IPC transdifferentiation capabilities of ASCs isolated from male and female tissues must be considered (Ogawa et al., 2004). Transdifferentiation of ASCs may be an opportunity to generate implantable IPC clusters that could restore natural, dynamic glucose regulation without the limitations, and concerns of other cell origins (Shapiro et al., 2000; Ryan et al., 2005).

Contemporary strategies to transdifferentiate adult MSCs into IPCs often results in immature, polyhormonal pancreatic cells (Li et al., 2013; Shivakumar et al., 2019; Wartchow et al., 2020). Current information supports the important role of mechanical cues, including fluid shear forces, to mechanotransduction that directs progenitor cell differentiation toward an endocrine phenotype, and biophysical cues are thought to guide differentiation of endocrine progenitors into mature *β* cells (Alessandra et al., 2020). Two-dimensional rocking of culture plates is an option to expose multiple individual samples to identical mechanical stresses during differentiation. Fluid shear stress is created in six well plates with 2-dimensional rocking motion. At a tilt angle of 7° and a frequency of 0.5 Hz, time-averaged shear stress is relatively stable at 0.033 Pa over the entire well, and both constant and interval exposure of human tenocytes cultured in monolayer increased cell collagen and glycosaminoglycan production over static culture (Tucker et al., 2014). Fluid shear forces and potential compressive shear forces from dynamic interactions between clusters with each other and cultureware surfaces during controlled rocking may enhance mechanotransduction and augment cell differentiation above that of static culture. De novo generation of functional IPC clusters *via* transdifferentiation of feline ASCs may not only provide a viable, functional cell therapy for feline DM, but may also serve as a platform for developing a comparable human treatment given feline and human DM similarities. Diabetes mellitus type II is most prevalent in both species, and the condition is associated with a combination of genetic and environmental factors (Osto et al., 2013). Both species also have a similar age at onset, association with obesity, diminished insulin secretion, islet amyloid deposition, and loss of approximately 50% of *β* cell mass (Henson and O'Brien, 2006). Microstructurally, feline islet morphology resembles that of human more closely than rodents with homogenous distribution of *α*, *β*, and *δ* cells in human and feline islets in contrast to peripheral distribution of *α* and *δ* cells with *β* cells concentrated at the islet center in rodents (Levetan and Pierce, 2013; Zini et al., 2016). The ability to generate viable, functional IPC clusters from ASCs, while technically different among species, may highlight a strategic therapeutic option for DM that capitalizes on MSC tissue generation capacity.

This study was designed to document the transdifferentiation capability of feline ASCs isolated from reproductive organ adipose tissue into IPC clusters and to characterize the *in vitro* characteristics and behavior of differentiated clusters with and without controlled rocking. The two-part hypothesis was that IPC clusters generated from fresh male and female ASCs have similar *in vitro* characteristics and behavior and that mechanical agitation improves maturation of cluster cells over static culture.

## Materials and Methods

### Study Design

**FIGURE 1:**
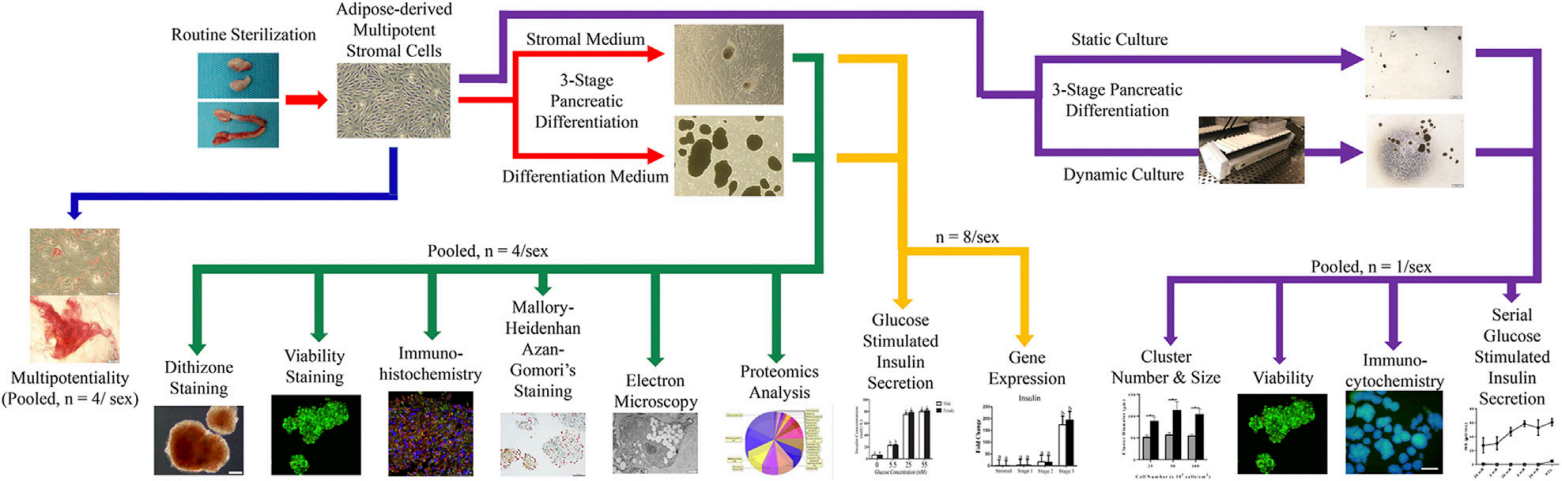
Study design schematic.

Feline ASCs were isolated according to a published protocol from adipose tissue collected during elective sterilization of eight male and eight female adult cats (Zhang et al., 2014). Plasticity was confirmed in cell passage (P) 1 with osteogenic and adipogenic differentiation. Cells were then mechanically induced to form clusters and subsequently exposed to a novel, three-stage culture process with stromal or IPC differentiation medium. Resulting clusters were evaluated for intracellular zinc with dithizone staining, viability with calcein/ethidium bromide stain, and intracellular insulin, glucagon, and somatostatin with immunohistochemistry and Mallory-Heidenhan Azan-Gomori’s stain. Ultrastructure was assessed with immunoelectron microscopy, and glucose stimulated insulin secretion in the presence or absence of theophylline was quantified with a feline-specific enzyme-linked immunosorbent assay (ELISA). Pancreatic lineage-specific gene and oncogene expression was quantified at each stage of differentiation using RT-PCR. A proteomics analysis was performed on clusters following liquid chromatography-mass spectrometric protein extraction (Figure 1).

To assess the effect of motion on differentiation, paired ASC samples from each donor were cultured stationary or with dynamic motion during the first two stages of the three-stage differentiation process. The resulting IPCs were evaluated for size, morphology, viability, intracellular insulin and glucagon, as well as serial glucose stimulated insulin secretion (Figure 1).

In this study, P0 is the first primary cell passage. Procedures performed at temperatures other than room temperature (20°C–25°C) are indicated. All materials and reagents were from Sigma-Aldrich, St. Louis, MO, United States unless noted otherwise.

### Cell Isolation

Adipose tissues were harvested from reproductive organs removed during elective sterilization, and cells were isolated as published with minor modifications (Zhang et al., 2014). Specifically, adipose tissues were minced and digested with 0.3% (≥375 units/ml) type I collagenase (Worthington Biochemical Corporation, Lakewood, NJ, United States) in Krebs-Ringer buffer (KRB) at 37°C for 30 min with 1 × 10^3^ rpm stirring. After filtering (100 μm nylon cell strainers, BD Falcon, Bedford, MA, United States) and centrifugation (260 × g, 5 min), the resulting stromal vascular fraction pellets were resuspended in 5 ml red blood cell lysis buffer (0.16 M NH_4_Cl, 0.01 M KHCO_3_, 0.01% ethylenediaminetetraacetic acid) for 5 min. The stromal vascular fraction was collected after centrifugation (260 × g, 5 min) and seeded in 10 cm cell culture dishes in stromal medium (Dulbecco’s modified Eagle’s medium F-12 (DMEM/F-12, Hyclone, Logan, UT, United States), 1% antibiotic/antimycotic solution (MP Biomedical, Irvine, CA, United States), 10% fetal bovine serum (FBS, Hyclone)). Stromal medium was refreshed after 24 h and then every 3 days. At 70%–80% confluence, the stromal vascular fraction cells were detached with 0.05% trypsin (Hyclone), and cells were seeded in T75 flasks at a density of 5 ×10^3^ cells/cm^2^ for P0 and all subsequent passages.

### Multipotentiality

Multipotentiality of P1 cells was confirmed as previously described (Zhang et al., 2014). Specifically, triplicate aliquots from each donor were cultured to 70%–80% confluence on 10 cm tissue culture plates (Nunc, Roskilde, Denmark) in stromal medium. One replicate was then fixed in 4% paraformaldehyde (PFA) and stained with 0.1% toluidine blue. The remaining two replicates were washed with phosphate buffered saline (PBS, Hyclone) and subsequently cultured in adipogenic induction medium (Table 1) for 10 days or osteogenic preinduction medium for 10 days followed by osteogenic induction medium (Table 1) for another 10 days. Cells were then fixed and stained with 4% PFA and oil red O (adipogenesis) or 70% ice cold ethanol and 2% alizarin red (osteogenesis). Stained colonies were imaged with a digital camera (DP21, Olympus, Shinjuku, Tokyo, Japan) on a light microscope (CKX41, Olympus).

**TABLE 1:** Differentiation media composition.

Medium	Composition
Adipogenic medium	Minimum essential medium alpha (α-MEM), 10% rabbit serum, 10% FBS, 10 nM dexamethasone, 5 μg/ml insulin, 50 μM 5,8,11,14-eicosatetraynoic acid (ETYA, Cayman Chemical, Ann Arbor, MI, United States), 100 μM indomethacin
Osteogenic preinduction medium	DMEM/F-12, 10% FBS, 100 nM dexamethasone, 0.25 mM L-ascorbic acid
Osteogenic induction medium	Osteogenic preinduction medium supplemented with 10 mM β-glycerophosphate
β-cell induction medium	Serum free medium (SFM) 1
	DMEM/F-12, 1% BSA, 1 × insulin-transferrin-selenium (ITS, Gibco, Gaithersburg, MD, United States), 4 nM activin A (R&D Systems., Minneapolis, MN, United States), 1 mM sodium butyrate, 50 μM 2-mercapethanol, 1% N-2 supplement (R&D Systems), 2% B-27 supplement (Gibco), 5 μg/ml laminin (Corning), 50 ng/ml recombinant human hepatocyte growth factor (HGF, EMD Millipore, Temecula, CA, United States), 20 ng/ml basic fibroblast growth factor (bFGF, Gibco)
	SFM 2
	DMEM/F-12, 1% BSA, 1 × ITS, 0.3 mM taurine (ACROS Organics, Morris Plains, NJ, United States), 5 μg/ml laminin, 20 ng/ml bFGF, 1% N-2 supplement, 2% B-27 supplement, 50 ng/ml HGF, 1 mM nicotinamide
	SFM 3
	DMEM/F-12, 1.5% BSA, 1.5 × ITS, 3 mM taurine, 100 nM glucagon-like peptide 1 (GLP-1, Tocris Bioscience, Ellisville, MO, United States), 1 mM nicotinamide (ACROS Organics), 1 × non-essential amino acids (NEAA, Gibco), 10 nM pentagastrin (Tocris Bioscience), 1% N-2 supplement, 1% B-27 supplement, 50 ng/ml HGF, 20 ng/ml bFGF, 5 μg/ml laminin, 20 ng/ml betacellulin (R&D Systems), 10 nM extendin-4 (Tocris Bioscience)

### Cell Culture and Differentiation

Isolated ASCs were cultured and expanded to P2 when they were detached as described above and then cultured in 24-well ultralow attachment plates (Corning, Corning, NY, United States) at 1 × 10^6^ cells/well for 1-2 days in stromal medium when cells formed clusters with a diameter of about 100 µm. A three-stage differentiation protocol was used for IPC cluster generation (Table 1). Clusters from 1 × 10^6^ cells/well were incubated with stage 1 medium for 2 days and then with stage 2 medium for 4 days in 24-well ultralow attachment plates. The clusters in each well were then transferred the wells of a standard 24-well plate and cultured in stage 3 medium for two more days with medium changes every day. At this step, some clusters attached to the plate surface while others remained detached. Those clusters that were not attached were decanted into a 15 ml conical tube, washed with PBS, centrifuged (350 × g, 8 min), resuspended in stage 3 medium, transferred to laminin-coated 24-well plates (Corning) and cultured for 4 days with medium changes every day, after which clusters were quantified and prepared for analysis. Parallel cultures of cells in stromal medium were treated identically to those in staged differentiation medium including cultureware and medium exchanges. Cluster aliquots for all assays were those clusters that formed in 1 well of a 24-well plate with an initial cell seeding density of 1 × 10^6^ cells/well.

### Dithizone Staining

Clusters were incubated with 10 μl dithizone (DTZ, Fisher Scientific, Fairlawn, NJ, United States) working solution (1 mg/ml DTZ in dimethyl sulfoxide (Fisher Scientific)) in 1 ml of stromal medium for 30 min at 37°C. They were decanted into a 15 ml conical tube, rinsed with PBS, transferred to a well of a 24-well culture plate, and imaged with a digital camera (DP21, Oympus) on an inverted light microscope (CKX41, Olympus).

### Viability Staining

Clusters were stained with viability stain (Live/Dead^™^, Invitrogen Corp., Carlsbad, CA, United States) according to the manufacturer’s instructions. Specifically, clusters were incubated with 1 ml of stain containing calcein-AM (viable) and ethidium homodimer-1 (non-viable) for 15 min in darkness at room temperature. Clusters were then washed with PBS and imaged with a digital camera (CLSM, Leica TCS SP2, Leica, Wetzlar, Germany) on a confocal laser scanning microscope to detect calcein-AM (515 nm) and ethidium homodimer-1 (635 nm) fluorescent emission after excitation (495 nm).

### RT-PCR—Gene Expression

Total RNA was isolated from clusters at each induction stage (EZNA^®^ MicroElute Total RNA kit, Omega Bio-Tek, Norcross, GA, United States). The quality and concentration were determined spectrophotometrically (NanoDrop ND-1000, NanoDrop Technologies, Wilmington, DE, United States), and cDNA synthesized (Maxima First-Strand cDNA synthesis kit, Thermo Fisher, Waltham, MA, United States). Expression of feline pancreatic target genes, insulin, ISL LIM homeobox 1 (Isl-1), hexokinase 1 (HK1), glucose transporter 2 (Glut-2), NK6 Homeobox 1 (Nkx6.1), nestin, neuronal differentiation 1 (Neurod1), ROS proto-oncogene 1 (ROS1), somatostatin (STS), glucagon (GCG), paired box 6 (Pax6), AKT serine/threonine kinase 1 (AKT1), and RAB3A (Table 2) were quantified with real-time RT-PCR using Thermo Fisher Absolute™ Blue QPCR Rox Mix technology and an ABI Prism 7900 HT Sequence Detection System (Applied Biosystems, Foster City, CA, United States). The 2^−ΔΔCt^ values were determined relative to the reference gene β-actin and presented as fold change normalized to clusters cultured in stromal medium.

**TABLE 2:** Feline primer sequences.

Gene		Sequence (5′–3′)	Accession number
β-actin	Forward	AGC​CTT​CCT​TCC​TGG​GTA​TG	XM_006941899.3
	Reverse	ACA​GCA​CCG​TGT​TAG​CGT​AG	
Nkx6.1	Forward	AACGAAATACTTGGCGG	XM_019829291.1
	Reverse	CCA​GAG​GCT​TGT​TGT​AGT​CG	
Pax6	Forward	GGCAATCGGTGGTAGTAA	XM_019812231.1
	Reverse	CTT​GGT​ATG​TTA​TCG​TTG​G	
Isl-1	Forward	CAAGGACAAGAAGCGGAG	XM_003981424.3
	Reverse	CTGGGTTTGCCTGTAAGC	
Glut-2	Forward	TTG​GCT​TGG​ATG​AGT​TAC​G	XM_003991916.3
	Reverse	GAC​TTT​CCT​TTG​GTT​TCC​G	
Nestin	Forward	ACC​CTG​ACC​ACC​CTA​GTT​TA	XM_045048569.1
	Reverse	GCA​GAC​CGT​TCA​CCA​TTT​T	
Neurod1	Forward	ACG​AAT​GTC​TCA​GTT​CTC​AGG	XM_003990912.5
	Reverse	CCT​CTT​CTT​CCT​CTT​CTT​CCA​G	
GCG	Forward	TGA​ACA​CCA​AGA​GGA​ACA​A	XM_006935320.2
	Reverse	ACCAGCCAAGCAATGAAT	
Insulin	Forward	CTTCGTCAACCAGCACC	XM_019811180.1
	Reverse	ACAGCATTGCTCCACGA	
STS	Forward	CCA​GAC​AGA​GAA​CGA​TGC​C	XM_003991805.4
	Reverse	CAG​GGT​TTG​AGT​TAG​TGG​A	
ROS1	Forward	AAC​AAC​AGC​CTC​TAC​TAC​AG	XM_019831130.1
	Reverse	TATCCTCCGACCGAATCC	
AKT1	Forward	CCA​ACA​CCT​TCA​TCA​TCC​G	NM_001322435.1
	Reverse	CCA​TCA​TTT​CCT​CCT​CCT​G	
HK1	Forward	TGA​GAA​GAT​GGT​GAG​TGG​C	XM_006937834.3
	Reverse	GGCAGAGCGAAATGAGAC	
RAB3A	Forward	TCCGCAACGACAAGAG	XM_034996482.1
	Reverse	AAG​AAC​TCA​AAG​CCA​AGG​TG	

### Immunohistochemistry

Cell clusters were fixed overnight in 4% PFA, embedded in paraffin, and sectioned (5 µm). Sections were blocked with 4% bovine serum albumin (BSA) in Tris-buffered saline (0.1% Tween-20, 20 mM Tris, 137 mM NaCl, pH = 7.6) for 30 min at room temperature. They were subsequently incubated with primary antibodies specific for feline antigens or validated for feline cross reactivity. Clusters were exposed to antibodies against insulin (1:1000), glucagon (1:2000), and somatostatin (1:1000) in pairs (insulin/somatostatin, glucagon/somatostatin) for 1 h at room temperature (Table 3). After a PBS wash, sections were incubated with secondary antibodies, goat anti-mouse IgG1-FITC (1:100) and goat anti-rabbit CF^™^−594 (1:500), for 1 h at room temperature. Nuclei were stained with 4’, 6-diamidino-2-phenylindole (DAPI). Photomicrographs were obtained with a digital camera (Leica TCS SP2, Leica) on a confocal laser microscope (Leica). Fluorescent images were captured at excitation 495 nm and emission 519 nm for insulin and glucagon and at excitation 593 nm and emission 614 nm for somatostatin. Sections were incubated with secondary antibodies alone and processed identically to quantify nonspecific binding.

**TABLE 3:** Study antibodies.

Antibody	Label	Marker expression	Manufacturer	Cat. No.	Species	Target species	Diluent
Insulin	N/A	*β* cell marker	Sigma-Aldrich	I2018	Mouse	Feline	PBS
Glucagon	N/A	*α* cell marker	Sigma-Aldrich	G2654	Mouse	Feline	PBS
Somatostatin	N/A	*δ* cell marker	Bio-Rad	8330–0154	Rabbit	Human	PBS
Goat anti-mouse IgG1	FITC	Secondary antibody	Bio-Rad	STAR132F	Goat	Mouse	PBS
Goat anti-rabbit IgG	CF^™^ 594	Secondary antibody	Sigma-Aldrich	SAB4600110	Goat	Rabbit	PBS
Goat anti-mouse IgG	10 nm gold	Secondary antibody	Electron Microscopy Sciences	25128	Goat	Mouse	PBS

### Mallory-Heidenhan Azan-Gomori’s Modification Assay

Paraffin sections prepared like those above were stained with 0.1% azocarmine G solution (Electron Microscopy Sciences, Hatfield, PA, United States) at 56°C for 1 h and then rinsed with distilled water. Stained sections were immersed in 1% aniline-alcohol (Electron Microscopy Sciences) for 30 min at room temperature and then rinsed with distilled water. They were transferred to iron alum solution (Electron Microscopy Sciences) for 5 min and then stained with aniline blue-orange G (Electron Microscopy Sciences) for 20 min. Light photomicrographs were obtained with a light microscope as before (Leica DM 4500b, Leica). The staining protocol results in orange to brown staining of β-like cells, bright red staining of α-like cells, and dark blue staining of δ-like cells.

### Glucose-Stimulated Insulin Secretion and Intracellular Content

To measure the response to distinct glucose levels, clusters from 2 × 10^6^ cells were collected in 15 ml conical tubes and incubated with 1 ml of KRB buffer for 4 h at 37°C. The supernatant collected after centrifugation (350 ×g, 8 min) was the baseline sample (0 mM glucose). Clusters were then suspended in 1 ml of KRB buffer supplemented with one of the following glucose concentrations, 5.5, 25, or 55 mM for 3 h each at 37°C. Two replicates of each sample were exposed to glucose concentrations in order from lowest to highest. Sample supernatants were collected after centrifugation (350 × g, 8 min) and stored in 1.5 ml microcentrifuge tubes (Fisher Scientific) at −80°C. The stored medium was thawed at room temperature and insulin levels quantified with a feline insulin ELISA kit (MyBioSource, San Diego, CA, United States) according to the manufacturer’s instructions.

To evaluate the effect of theophylline on insulin secretion, cell clusters were incubated in 1 ml of KRB buffer with 5.5 mM glucose for 3 h at 37°C. Clusters were then incubated with 1 ml of KRB buffer with either 25 or 55 mM glucose in the presence or absence of 5 mM theophylline (MP Biomedicals) for 1 h at 37°C. Insulin fold change was calculated relative to the 5 mM glucose sample without theophylline as: (Insulin level at 25 or 55 mM glucose with or without theophylline)/(Insulin level at 5.5 mM glucose).

Additionally, to quantify the effects of theophylline on intracellular insulin, paired clusters were incubated in 1 ml of KRB buffer with 55 mM glucose in the presence or absence of 5 mM theophylline for 1 h at 37°C. Subsequently, intracellular insulin was extracted from clusters using an established acid-ethanol method (Carlsson et al., 2010). Briefly, cell clusters were centrifuged (350 × g, 8 min), washed with PBS, and incubated with 100 µl acid-ethanol (1.5% HCl in 70% ethanol) at −20°C overnight. The mixture was centrifuged (2.1 × 10^4^ × g) at 4°C for 20 min, followed by collection of supernatant, and then 100 µl 1 M Tris in water (pH = 7.5) was added. The fold change in insulin levels was calculated as: (Insulin level at 55 mM glucose with theophylline)/(Insulin level at 55 mM glucose without theophylline).

### Transmission Electron Microscopy Immunolabelling

Clusters were rinsed with PBS and then fixed in 2% PFA and 2.5% glutaraldehyde in 0.1 M PBS for 10 min. After centrifugation at (1 × 10^3^ × g) in 1.5 ml microcentrifuge tubes for 10 min, the supernatant was removed, and the samples were again fixed in 2% PFA and 2.5% glutaraldehyde in 0.1 M PBS with agitation for 2 h at room temperature. Samples were mixed with equal amounts of 3% agarose and placed on a glass slide (Leica S9E, Leica). The solidified mixture was cut into cubes which were placed into a glass vial filled with 0.1 M PBS. Cubes were then washed with 0.1 M PBS and 0.08 M glycine five times, 15 min each, followed by fixation in 2% osmium tetroxide in 0.1 M PBS for 1 h in darkness. Samples were washed with H_2_O and dehydrated in a series of ethanol-distilled water solutions. Dehydrated samples were infiltrated with 1:1 ethanol and LR white resin for 2 h, and then infiltrated with 100% LR white resin for another 2 h. Embedded samples were incubated at 18°C for 24 h. Ultra-thin sections (90 nm) were stained with 2% uranyl acetate and lead citrate and images generated with a TEM (JEM-1400, JEOL Ltd., Akishima, Tokyo, Japan) imaging system. For immunolabeling, sections were incubated in 5% BSA in PBS for 30 min and then with a mouse anti-insulin antibody (1:20, Table 3) in 1% BSA in PBS for another 90 min at room temperature. After incubation, the sections were washed with 1% BSA buffer and PBS. Sections were then incubated with goat anti-mouse secondary antibody labeled with 10 nm gold particles (1:50) (Electron Microscopy Sciences) in 1% BSA in PBS for 90 min. After incubation, sections were washed with 1% BSA buffer and PBS. The sections were then fixed in 2% glutaraldehyde in PBS for 5 min and strained with 2% uranyl acetate and lead citrate after being thoroughly washed in distilled water. Sections were imaged with a high-resolution water-cooled CCD-camera on a TEM (JEM-1400, JEOL Ltd.).

### Scanning Electron Microscopy

Clusters were fixed in 2% PFA and 2% glutaraldehyde in 0.1 M PBS on a 13 mm diameter 2 μm pore polycarbonate filter. They were then rinsed with 0.1 M PBS and distilled water. Samples were sequentially dried with hexamethyldisilazane (HMDS, Electron Microscopy Sciences), 1:1 100% ethanol and HDMS, and 100% HDMS. After HDMS evaporation, samples were coated with platinum in an EMS 550X sputter coater (Electron Microscopy Sciences) and imaged with a scanning electron microscope camera (JSM-6610, JEOL Ltd.) under high vacuum mode.

### Liquid Chromatography–Mass Spectrometry Proteomic Analysis

Proteins were reduced, alkylated, and purified by chloroform/methanol extraction prior to digestion with sequencing grade modified porcine trypsin (Promega, Madison, WI, United States). Tryptic peptides were then separated by reverse phase XSelect CSH C18 2.5 μm resin (Waters, Milford, MA, United States) on an inline 150 mm × 0.075 mm column using an UltiMate 3000 RSLCnano system (Fisher Scientific). Peptides were eluted using a 90 min gradient from 98:2 to 65:35 buffer A (0.1% formic acid, 0.5% acetonitrile): B (0.1% formic acid, 99.9% acetonitrile) ratio. Eluted peptides were ionized by electrospray (2.2 kV) followed by mass spectrometric analysis (Orbitrap Fusion Lumos, Fisher Scientific). MS data were acquired using the FTMS analyzer in profile mode at a resolution of 120,000 over a range of 375–1,500 m/z. Following HCD activation, MS/MS data were acquired using the ion trap analyzer in centroid mode and normal mass range with precursor mass-dependent normalized collision energy between 28.0 and 31.0. Proteins were identified with a database search using MaxQuant (Max Plank Institute of Biochemistry, Martinsried, Germany) with a parent ion tolerance of 3 ppm and a fragment ion tolerance of 0.5 Da. Scaffold Q + S (Proteome Software, Inc., Portland, OR, United States) was used to verify MS/MS based peptide and protein identifications which were accepted if they could be established with less than 1.0% false discovery and contained at least two identified peptides. Protein probabilities were assigned by the Protein Prophet algorithm (Nesvizhskii et al., 2003). Identified proteins were categorized based on gene ontology (GO) terms using Scaffold Q + S.

### Mechanical Agitation Culture

To assess the effect of mechanical agitation during differentiation, paired cell aliquots from two donors were cultured with and without continuous cultureware motion through stage 2 of the three stage differentiation process. Cells at initial seeding densities of 2.5 × 10^4^ cells/cm^2^, 5.0 × 10^4^ cells/cm^2^, and 1.0 × 10^5^ cells/cm^2^ in 24-well plates were cultured in differentiation medium as described above while static or with two-dimensional rocking at 17 rpm (Vari-Mixer™ test tube rocker, Thermo Fisher). For stage 3 of the differentiation process, clusters were cultured in 24-well laminin-coated plates without rocking. Images were obtained at the completion of the differentiation process using a light microscope (CKX41, Olympus) equipped with a digital camera (DP21, Oympus). Cluster diameters and number/well were determined for all samples (ImageJ, National Institutes of Health, Bethesda, MD, United States). Subsequently, two paired aliquots of clusters generated from 2.5 × 10^4^ cells/cm^2^ (4.75 × 10^4^ cells/well) in a 24-well plate were cultured and differentiated identically to above, and cell viability, immunocytochemical localization of intracellular insulin and glucagon, and serial glucose stimulated insulin secretion assessed. Cell viability staining was determined identically to above.

For immunocytochemical labeling, IPC clusters were fixed in 4% PFA for 10 min at room temperature. After washing in PBS, clusters were permeabilized with 0.1% Triton^™^ X-100 (Thermo Fisher) for 10 min at room temperature. Clusters were blocked with 1% BSA and glycine (22.5 mg/ml) in PBS supplemented with 0.1% Tween-20 (Amresco Biochemicals, Solon, OH, United States) for 30 min at room temperature. They were then exposed to antibodies against insulin (1:1000) and somatostatin (1:1000) overnight at 4°C (Table 3). After a PBS wash, clusters were incubated with secondary antibodies, goat anti-mouse IgG1-FITC (1:100), and goat anti-rabbit CF-594^™^ (1:500), for 1 h at room temperature. Nuclei were stained with DAPI. Photomicrographs were obtained with a digital camera (Leica MC170 HD, Leica) on a fluorescent microscope (Leica DMIL LED, Leica). Fluorescent images were captured at 495 nm excitation and 519 nm emission for insulin and at 593 nm excitation and 614 nm emission for somatostatin.

Serial glucose stimulated insulin secretion was assessed in clusters generated from approximately 1 × 10^6^ cells collected from 24 wells initially seeded at 2.5 × 10^4^ cells/cm^2^. Clusters were incubated with 1 ml of KRB buffer for 4 h at 37°C in 15 ml conical tubes. The supernatant collected after centrifugation (350 × g, 8 min) was the baseline sample (0 mM glucose). Clusters were then incubated in 1 ml 20 mM (high) and 2 mM (low) glucose in KRB buffer for 30 min at 37°C for a total of three times at each concentration followed by a final incubation in 1 ml of low glucose in KRB buffer with potassium chloride (30 mM). After each 30 min incubation period, the sample supernatant was collected following centrifugation (350 × g, 8 min) and stored in 1.5 ml microcentrifuge tubes (Fisher Scientific) at −80°C. Between each incubation, clusters were washed with KRB buffer and centrifuged three times (350 × g, 8 min). The stored medium was thawed at room temperature and insulin levels quantified in two replicates of each sample with a feline insulin ELISA kit (MyBioSource) according to the manufacturer’s instructions.

### Statistical Analysis

All results are presented as least squares (LS) mean ± SEM. Statistical analyses were performed with commercially available software (JMP v13.0.0, SAS Institute Cary, NC, United States). Mixed ANOVA models were used to evaluate insulin fold change with fixed effects of glucose concentration and sex and a random effect of donor. The same models were used to evaluate target gene expression with fixed effects of induction stage and sex and a random effect of donor. Tukey’s post hoc tests were applied for multiple group comparisons (*p* < 0.05).

## Results

### Multipotentiality

**FIGURE 2:**
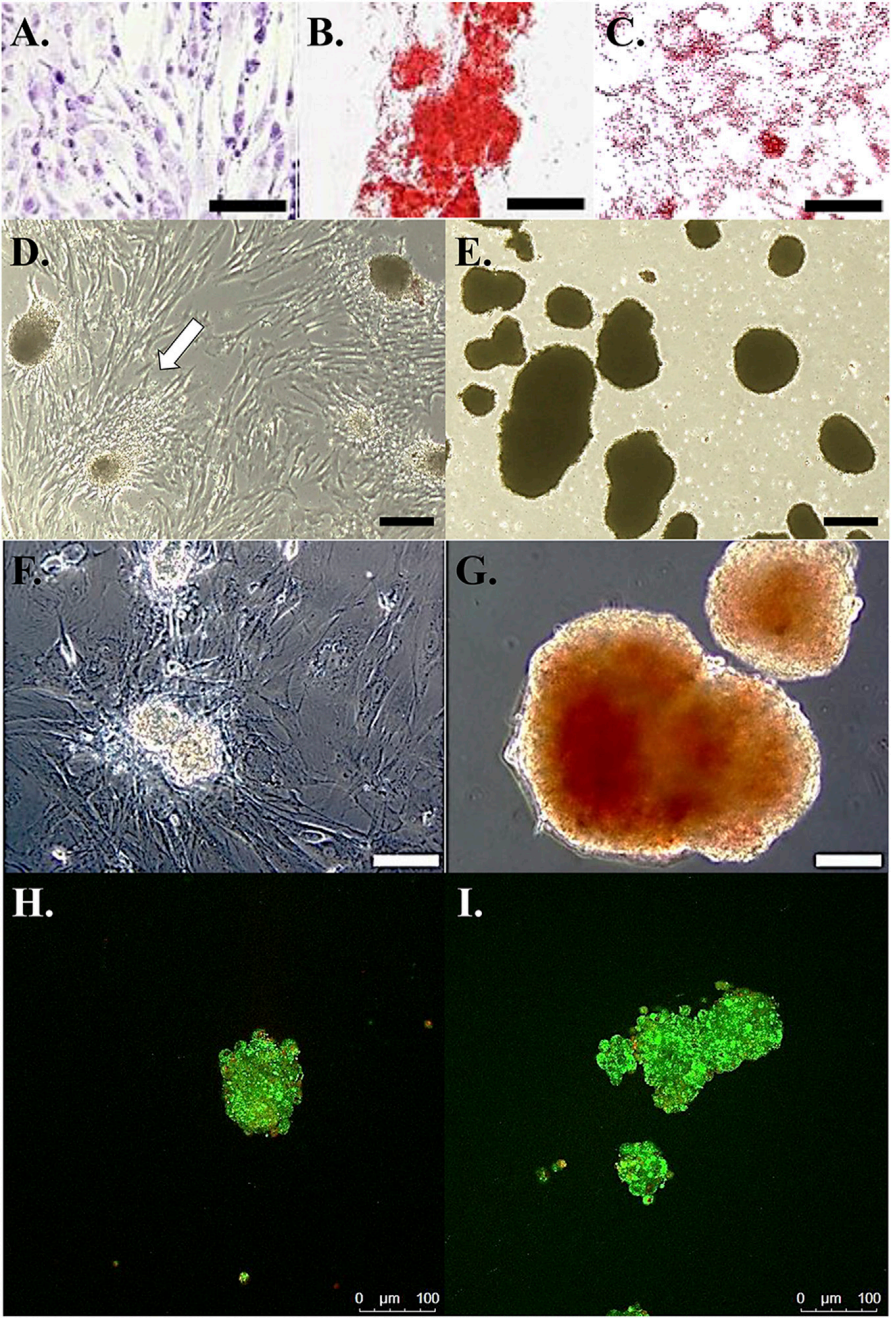
Photomicrographs of feline ASCs following culture in stromal medium **(A)**, with calcium deposition alizarin red staining after culture in osteogenic medium **(B)**, and with oil red O lipid staining after culture in adipogenic medium **(C)**. Light photomicrographs of cell clusters from feline ASCs in polystyrene cultureware after 3-stage culture in stromal **(D)** or pancreatic induction **(E)** medium. Cells attached to the polystyrene surface are evident with clusters cultured in stromal medium (arrow). Dithizone staining of zinc [red, **(F,G)**] and viability staining **(H,I)** of viable (green) and non-viable (red) cells in clusters from feline ASCs after 3-stage culture in stromal **(F,H)** or pancreatic induction **(G,I)** medium. Scale bars = 200 μm **(A,D,E)**, 50 μm **(B,C)**, and 100 μm **(F–I)**.

Cells cultured in stromal medium maintained an elongated, ovoid to stellate morphology in loosely organized colonies (Figure 2A). Those cultured in osteogenic medium formed tightly organized colonies that contained alizarin red staining calcium deposits (Figure 2B). Intracellular lipid droplets stained red with oil red O after culture in adipogenic medium (Figure 2C).

### Cell Culture and Differentiation

After transfer to a standard culture plate prior to stage 3, cell clusters cultured in stromal medium adhered to the plate, and cells migrated from the clusters to the plate surface within 24 h (Figure 2D). Those clusters cultured in induction medium remained detached at stage 3 (Figure 2E).

### Dithizone Staining

Cell clusters cultured in induction medium stained with DTZ (Figure 2G), and those cultured in stromal medium did not (Figure 2F).

### Viability Staining

The majority of cells within clusters cultured in stromal or induction medium were viable up to a cluster diameter of about 100 µm (Figures 2H,I). Cell viability tended to decrease with increasing diameter in clusters over 100 µm in diameter with nonviable cells concentrated near cluster centers.

### RT-PCR—Gene Expression

**FIGURE 3:**
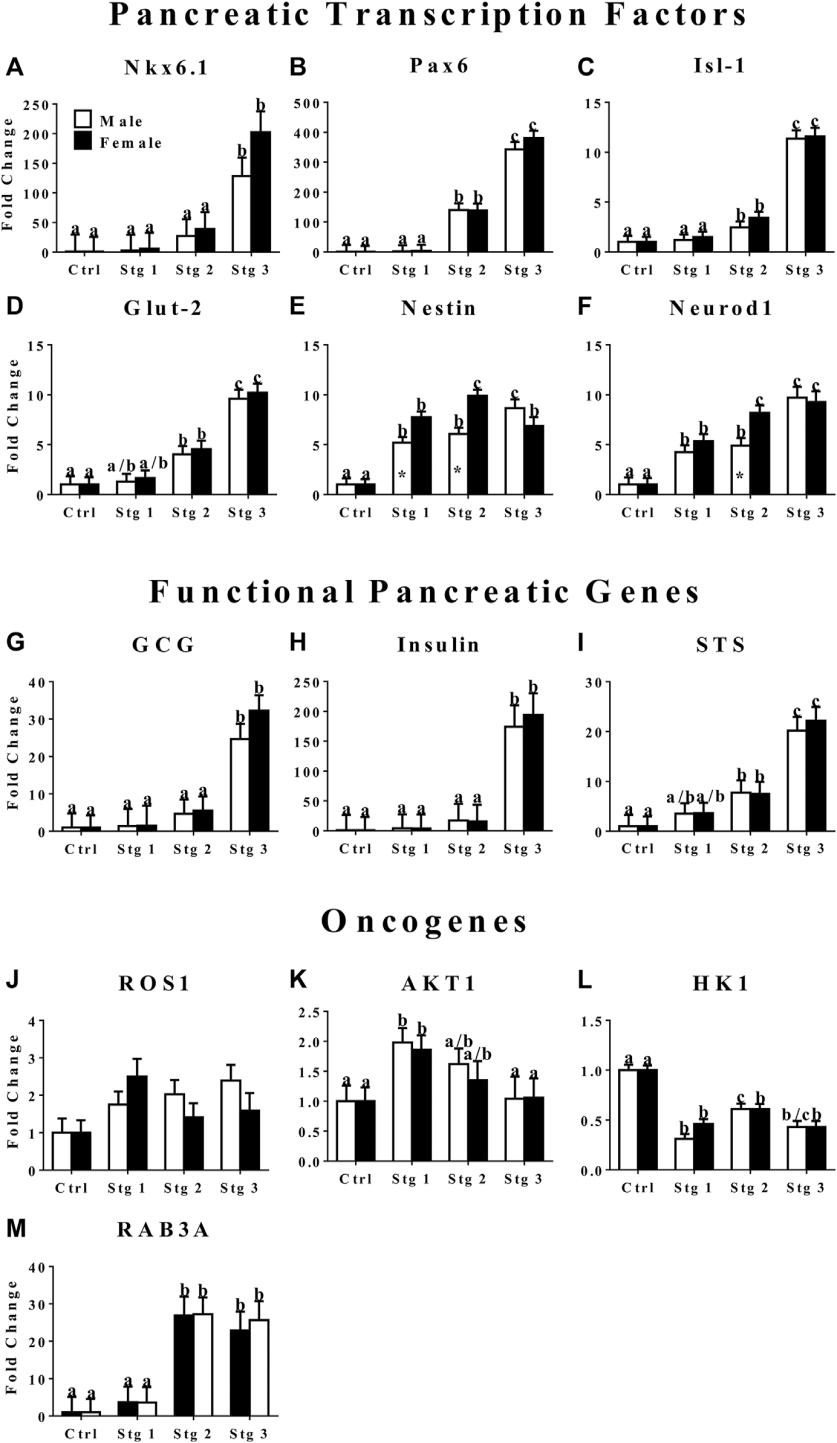
Fold change (2^−ΔΔCt^ , LS mean ± SEM) of feline transcription factor, NK6 homeobox 1 [Nkx6.1, **(A)**], paired box 6 [Pax6, **(B)**], ISL LIM homeobox 1 [Isl-1, **(C)**], glucose transporter 2 [Glut-2, **(D)**], nestin **(E)**, neurogenic differentiation factor 1 [Neurod1, **(F)**]; functional pancreatic, glucagon [GCG: **(G)**], insulin **(H)**, somatostatin [STS: **(I)**]; and oncogene, tyrosine-protein kinase ROS1 [ROS1: **(J)**], AKT serine/threonine kinase 1 [AKT1: **(K)**], hexokinase 1 [HK1: **(L)**], and Ras-related protein Rab-3A [RAB3A: **(M)**] expression levels by feline ASCs after culture in three stages (stg 1,2,3) of pancreatic differentiation media or stromal medium (Ctrl). Columns with distinct superscripts are significantly different among induction stages within sexes and those with asterisks are different between sexes within induction stages (*p* < 0.05).

The mRNA levels of transcription factors Nkx 6.1, Pax6, Isl1, and Glut-2 were highest at stage 3 of induction (Figure 3). Cells at stage 2 of induction had greater Pax6 and Isl-1 expression than those at stage 1 (Figures 3B,C). Nestin, a stemness marker, expression in cells from male donors increased with induction stage with highest expression at induction stage 3, female donor cells had the highest expression at stage 2 with similar expression between induction stages 1 and 3 (Figure 3E). Female donor cells had higher nestin expression at induction stages 1 and 2 compared to cells from male donors (Figure 3E). Neurod1, a transcription factor that plays an important role in *β* cell maturation, expression increased with induction stage in cells from both donor sexes with the greatest expression at stage 3 for cells from male donors and at stage 2 and 3 for cells from female donors (Figure 3F). Cells from female donors had greater Neurod1 expression compared to male donors at induction stage 2. The expression of GCG, insulin, and STS, markers for functional *α*, *β*, and *δ* cells, respectively, increased with induction stage and was highest at induction stage 3 (Figures 3G–I). In contrast, the oncogene AKT1 expression in cells from both sexes decreased with increasing induction stage with the highest expression at induction stage 1 (Figure 3K). The expression of HK1, another oncogene, remained significantly lower at all induction stages in cells cultured in induction medium compared to cells cultured in stromal medium (Figure 3L). Cells from male donors had greater HK1 expression at induction stage 2 versus 1 (Figure 3L). The expression of oncogene RAB3A was greater at induction stages 2 and 3 versus 1 in cells from both sexes (Figure 3M).

### Immunohistochemistry

**FIGURE 4:**
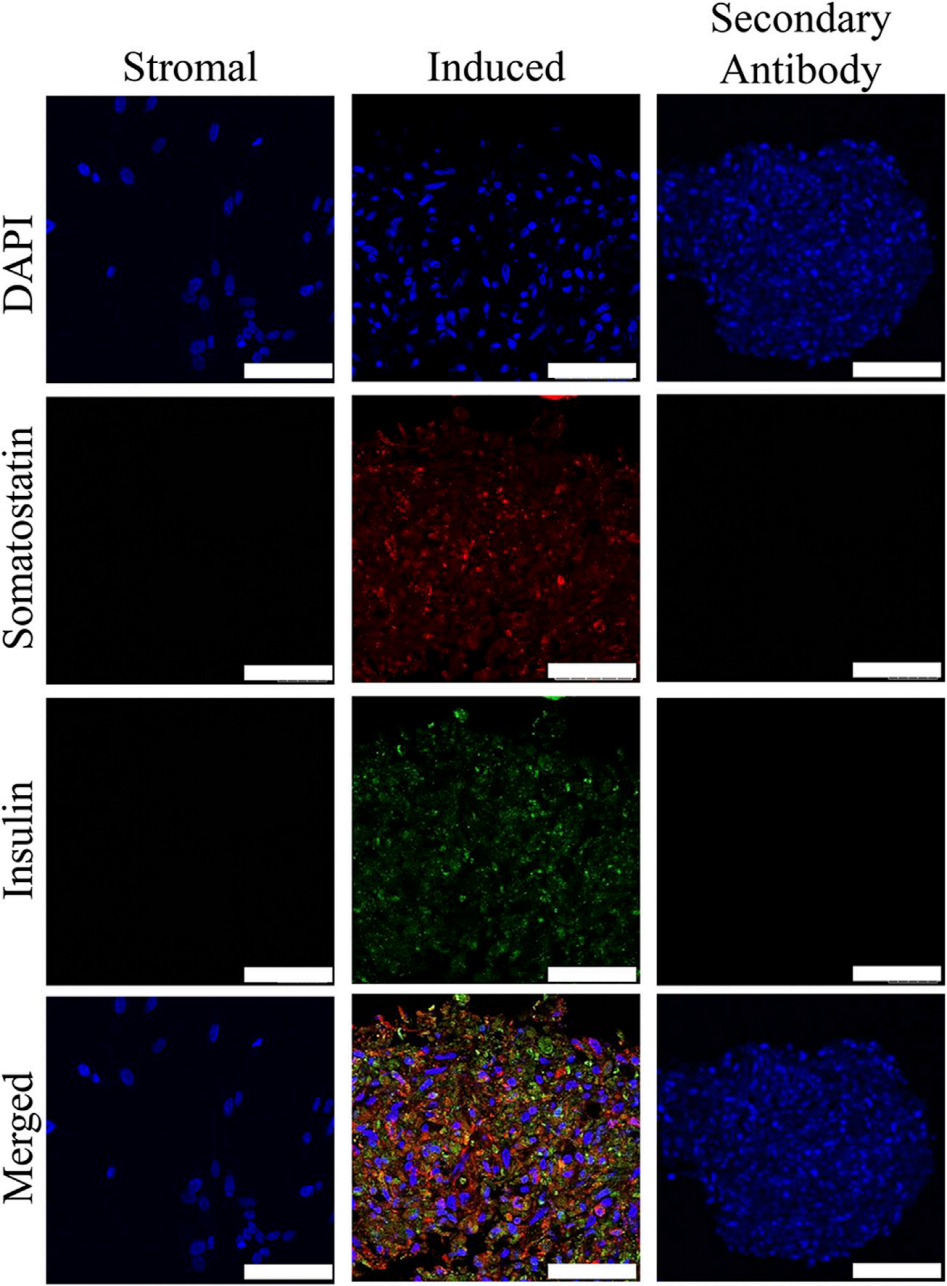
Fluorescent photomicrographs of cell clusters after culture in stromal medium (Stromal) or three stages of pancreatic induction medium (Induced) from pooled male and female feline ASCs labeled with DAPI (blue), somatostatin (red) and insulin (green). Clusters labeled with only the secondary antibody (Secondary Antibody) are also shown. Scale bars = 50 µm.

**FIGURE 5:**
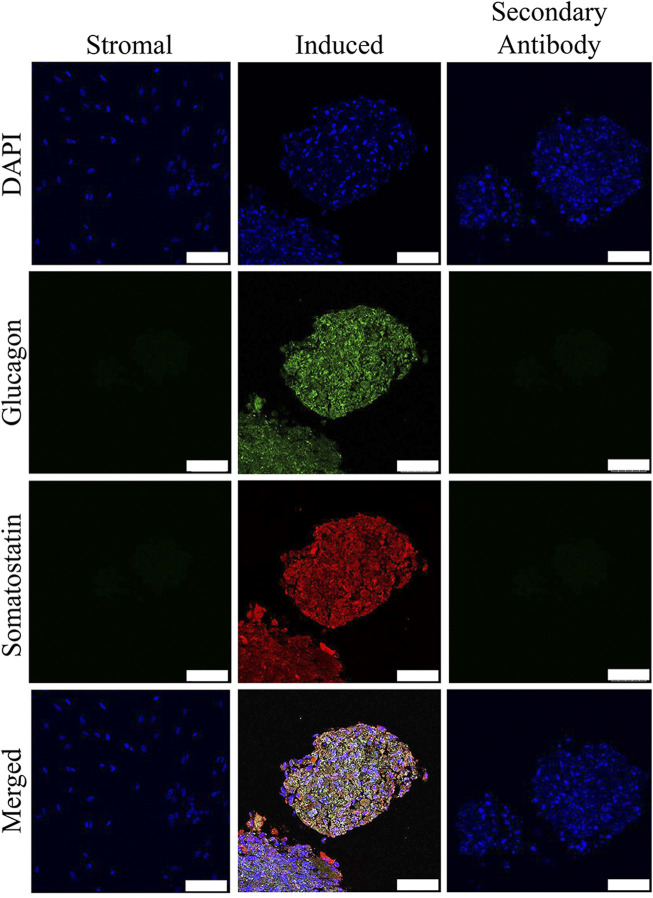
Fluorescent photomicrographs of cell clusters after culture in stromal medium (Stromal) or three stages of pancreatic induction medium (Induced) from pooled male and female feline ASCs labeled with DAPI (blue), somatostatin (red) and glucagon (green). Clusters labeled with only the secondary antibody (Secondary Antibody) are also shown. Scale bars = 50 µm.

Insulin and somatostatin as well as glucagon and insulin were localized within clusters cultured in induction medium while none was present in clusters cultured in stromal medium (Figures 4, 5).

### Mallory-Heidenhan Azan-Gomori’s Modification Assay

**FIGURE 6:**
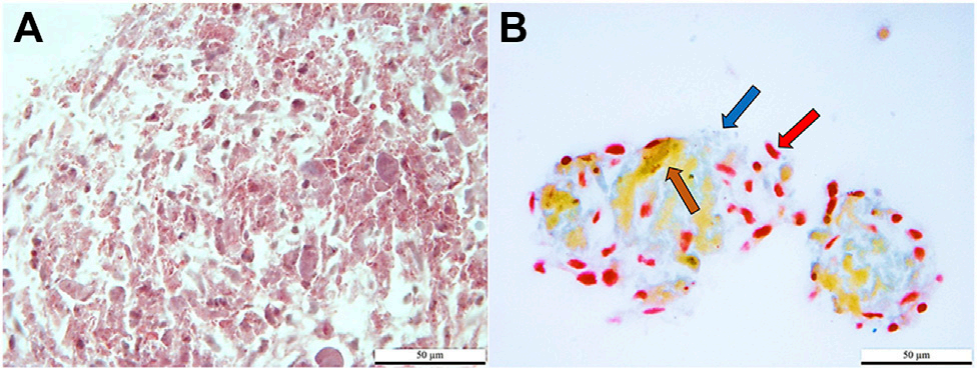
Light photomicrographs of feline ASCs after three-stage culture in stromal **(A)** or pancreatic induction **(B)** medium stained with Mallory-Heidenhan Azan-Gomori’s modification stain [*α* cell granules (red arrow): red; *β* cell granules (brown arrow): orange-brown; *δ* cell granules (blue arrow): blue]. Scale bars = 50 µm.

Cells stained red, brown, and dark blue, consistent with the presence of *α*, *β*, and *δ* cells in clusters cultured in induction medium. Clusters cultured in stromal medium had light red, non-specific staining (Figure 6).

### Glucose-Stimulated Insulin Secretion

**FIGURE 7:**
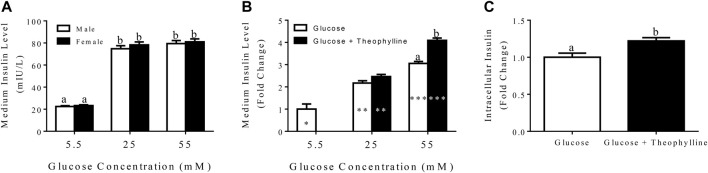
Medium insulin levels (LS mean ± SEM) following incubation of differentiated clusters with KRB buffer containing distinct glucose concentrations **(A)**. Fold change (LS mean ± SEM) in medium insulin levels relative to 5.5 mM glucose following incubation of differentiated clusters with KRB buffer containing distinct glucose concentrations with and without theophylline **(B)**, and intracellular insulin levels (LS mean ± SEM) following cluster incubation in KRB buffer containing 55 mM glucose with (black bar) and without (white bar) theophylline **(C)**. Columns with distinct superscripts are significantly different between theophylline treatments within glucose concentrations, and those with different asterisk (*) numbers are significantly different among glucose concentrations within theophylline treatments (*p* < 0.05).

In general, insulin secretion increased with increasing glucose concentration, and the highest secretion was obtained following incubation in medium containing 55 mM glucose for 3 h for both male and female donors (Figure 7A).

After incubation in medium with 25 mM glucose for 3 h, the fold increase of secreted insulin was higher compared to incubation in medium with 5.5 mM glucose and lower than incubation in 55 mM glucose regardless of theophylline treatment (Figure 7B). In contrast, in medium with 55 mM glucose, insulin secretion was higher with versus without theophylline treatment (Figure 7B). Additionally, intracellular insulin increased when clusters were cultured in medium with 55 mM glucose theophylline over no theophylline (Figure 7C).

### Transmission Electron Microscopy Immunolabelling

**FIGURE 8:**
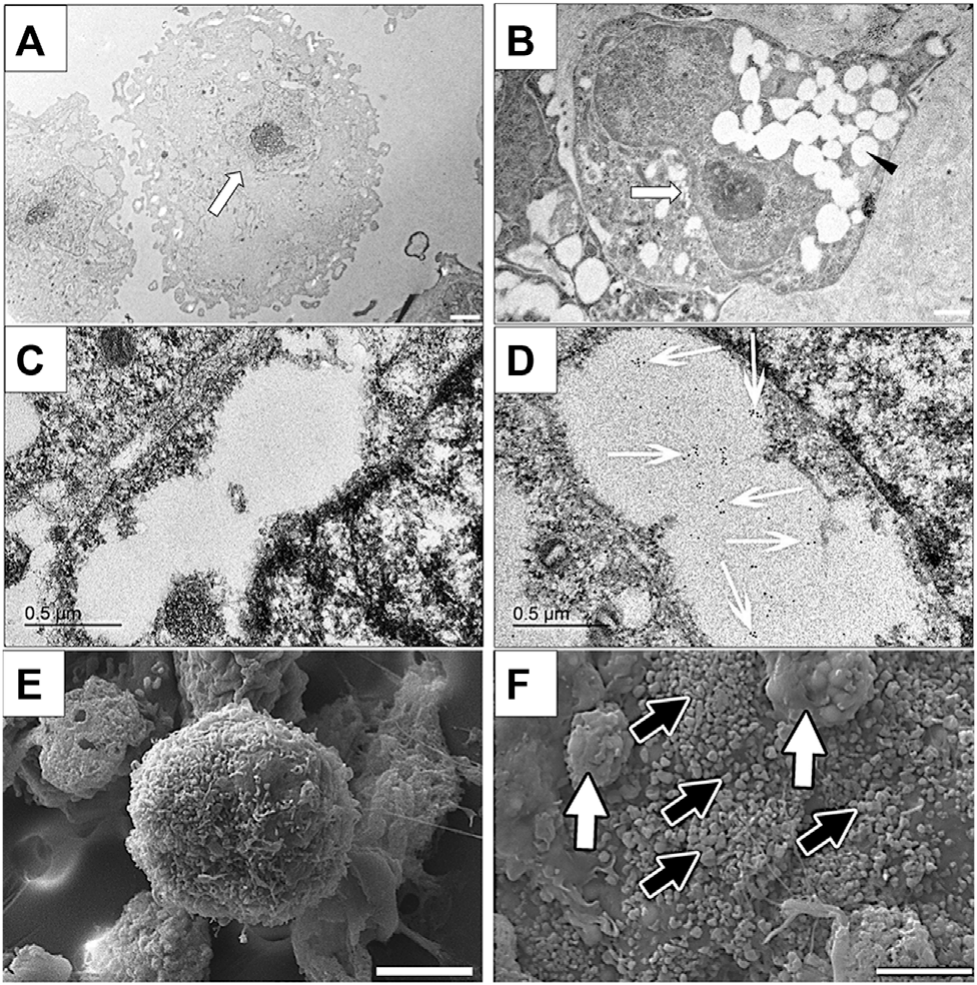
Transmission electron photomicrographs **(A,B)**, immunoelectron photomicrographs **(C,D)** and scanning electron photomicrographs of cells in feline ASC clusters following three stages of culture in stromal **(A,C,E)** or differentiation **(B,D,F)** medium. Nuclei [**(A,B)** white arrow] and vacuoles [**(B)**, black arrowhead], gold-labeled insulin within a vacuole [**(D)**, white arrows], and individual [black arrows, **(F)**] and clumped [white arrows, **(F)**] surface granules are indicated. Scale bars = 2 μm **(A)**, 1 μm **(B)**, 0.5 μm **(C,D)**, and 5 μm **(E,F)**.

Clusters cultured in differentiation medium contained cells that were characterized by numerous cytoplasmic vesicles and a proportionately smaller, lobed nucleus compared to those cultured in stromal medium that did not contain vesicles and had a large, irregular, round nucleus (Figures 8A,B). Immunoelectron microscopy confirmed the presence of insulin within vacuoles only in cells within clusters cultured in differentiation medium (Figures 8C,D).

### Scanning Electron Microscopy

Cell clusters cultured in differentiation medium were larger and composed of tightly adhered cells that had numerous globular surface deposits, consistent with secretory granules, in contrast to those cultured in stomal medium which were smaller, and composed of loosely associated cells with rare surface deposits (Figures 8E,F).

### Liquid Chromatography–Mass Spectrometry Proteomic Analysis

**FIGURE 9:**
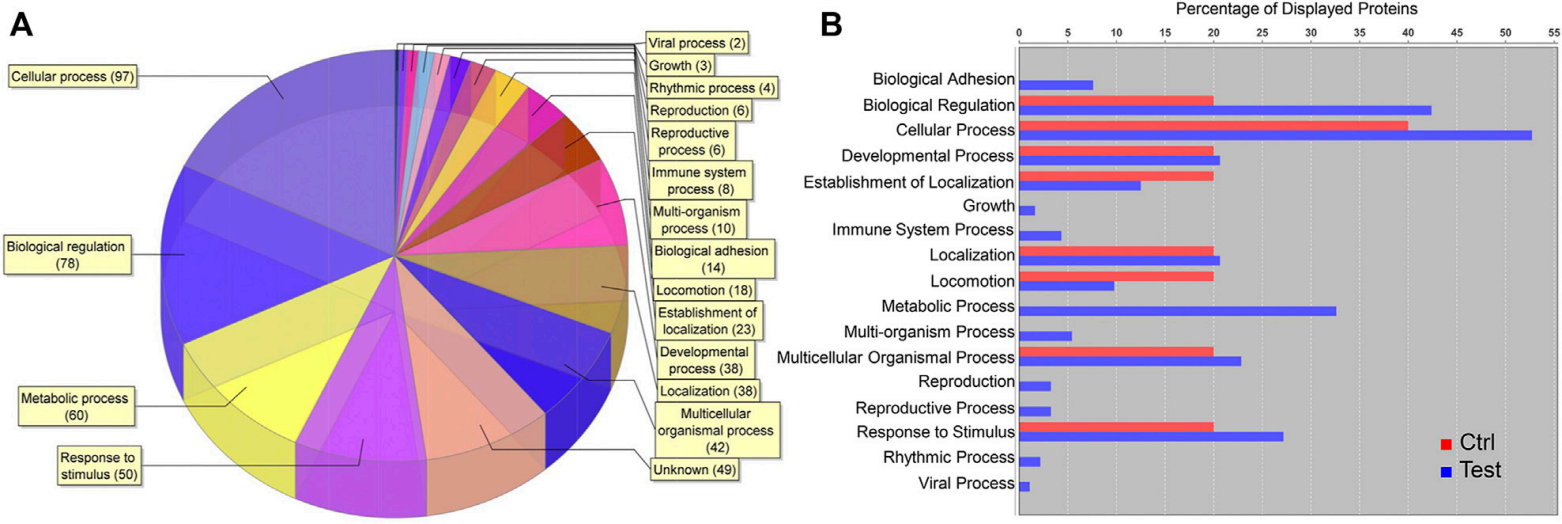
Proteomic analysis of cell clusters from pooled male and female feline ASCs following culture in pancreatic differentiation (Test) or stromal (Ctrl) medium. A total of 180 proteins were upregulated in the clusters, and the relative distribution among protein GO terms is shown **(A)** as well as the percentages of each in differentiated (blue) and undifferentiated (red) clusters **(B)**.

Among 2,516 total proteins identified, 180 were upregulated, 425 downregulated, and 1911 unchanged in cell clusters cultured in differentiation versus stromal medium. Among upregulated proteins, cellular processes, biological regulation, metabolic processes, and response to stimulus comprised most GO terms (Figure 9A). Upregulation of proteins within the metabolic process category occurred only in clusters cultured in differentiation medium (Figure 9B).

### Mechanical Agitation Culture

**FIGURE 10:**
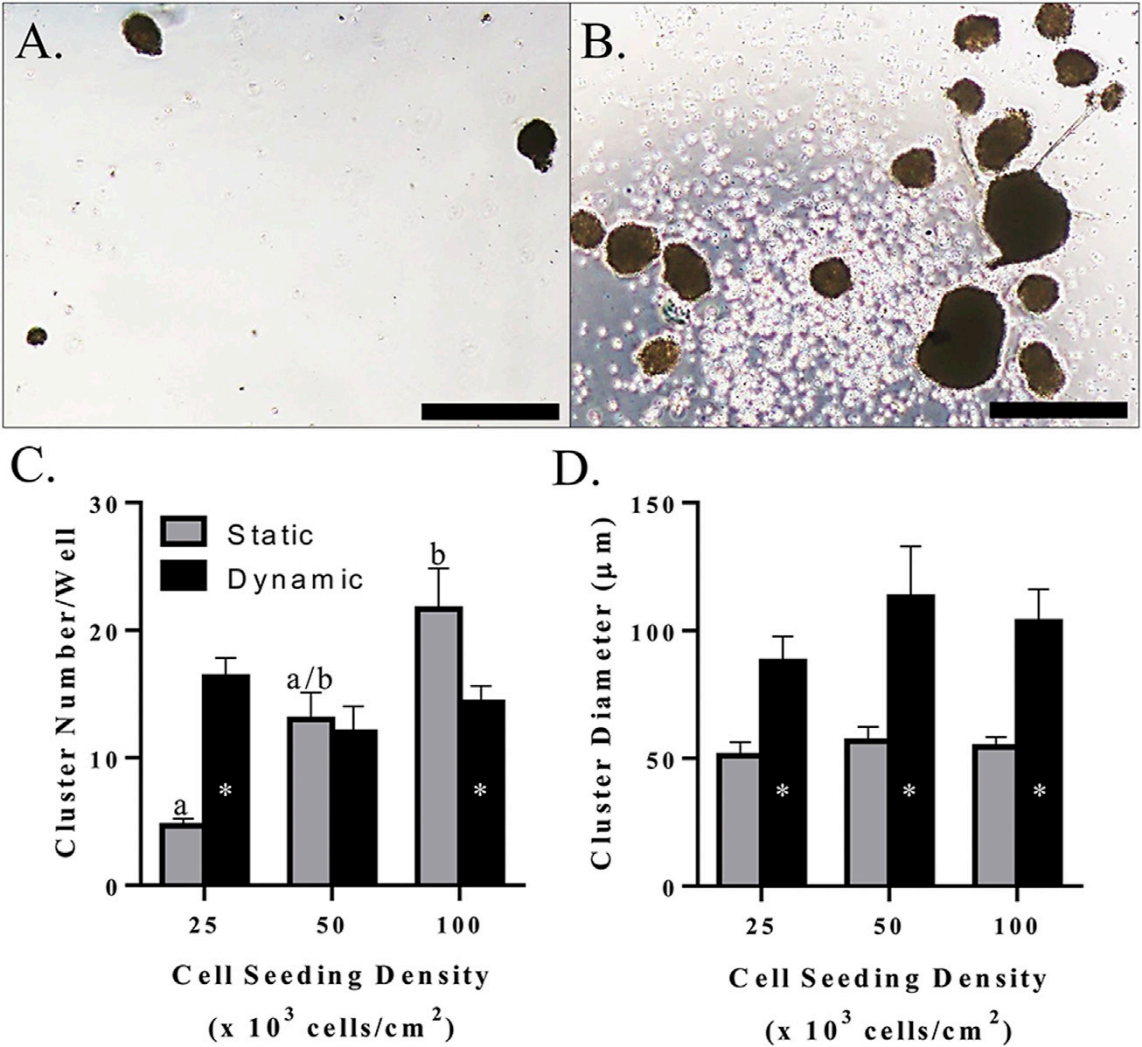
Light photomicrographs of feline ASC clusters **(A,B)**, LS mean ± SEM cluster number/well **(C)**, and cluster diameter **(D)** following pancreatic differentiation without **(A)** and with **(B)** dynamic culture during the first two stages of differentiation following culture in three-stage differentiation medium. Scale bars = 200 μm. Columns with distinct superscripts are significantly different among cell seeding densities within each culture condition, and columns with different asterisk numbers are significantly different between static and dynamic culture conditions within seeding densities (*p* < 0.05).

Morphologically, clusters were spherical and had a smooth surface when cultured without mechanical agitation, whereas those produced with dynamic culture tended to have an irregular shape and rough surface (Figure 10A,B). The number of clusters increased with increasing initial cell seeding density with static culture, but a total number of about 15/well in a 24-well plate remained constant with mechanical agitation at all seeding densities tested in this study (Figure 10C). This resulted in a higher cluster number with mechanical agitation at an initial density of 2.5 × 10^4^ cells/cm^2^ and a higher cluster number with static culture at an initial seeding density of 1.0 × 10^5^ cells/cm^2^ (Figure 10C). Regardless of seeding density, clusters had a diameter of about 100 μm with mechanical agitation which was significantly higher than clusters cultured statically with a diameter of about 50 μm (Figure 10D).

**FIGURE 11:**
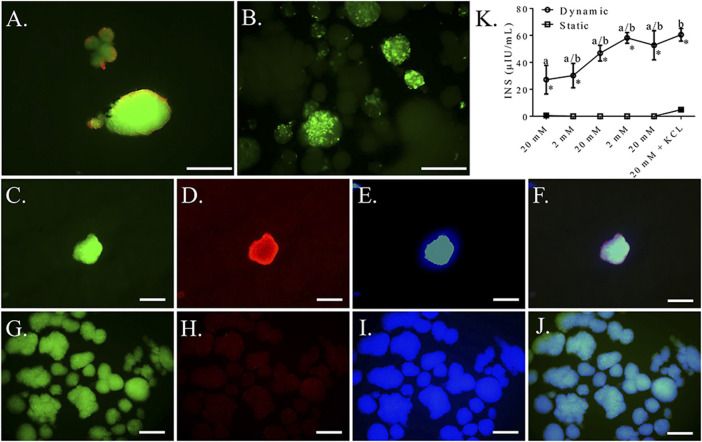
Fluorescent photomicrographs of feline ASC clusters cultured following culture in pancreatic differentiation medium without **(A,C–F)** and with **(B,C–J)** dynamic culture and stained with viability stain [**(A,B)**, viable—green, non-viable—red] or anti-insulin [**(C,G)**, green], anti-glucagon [**(D,H)**, blue], or DAPI [**(E,I)**, blue] separately and with images merged **(F,J)**. Scale bars = 100 μm. Insulin secretion (INS) by the same clusters cultured without (Static) and with (Dynamic) mechanical agitation following exposure to alternating low (2 mM) and high (20 mM) glucose concentrations for 30 min each followed by depolarization with potassium chloride (KCL) in low glucose buffer **(K)**. Data points with distinct superscripts are significantly different among incubation periods within culture condition and different asterisk (*) numbers indicate differences between static and dynamic culture conditions glucose concentration (*p* < 0.05).

Based on the above results, a seeding density of 2.5 × 10^4^ cells/cm^2^ was used for the remaining dynamic culture evaluations. Cells formed spheroids of viable cells with a mean diameter of 87.88 ± 9.93 μm (Figures 11A,B). Clusters cultured with mechanical agitation expressed insulin and glucagon, though insulin expression appeared to be the predominate of the two and glucagon expression appeared to be lower than in clusters with static culture (Figures 11C–J). Insulin secretion from clusters cultured with mechanical agitation tended to increase with each alternating high and low glucose stimulus, though insulin secretion was only statistically different between the first high glucose stimulus and the last low glucose stimulus combined with potassium chloride (Figure 11K). Clusters cultured with mechanical agitation had significantly higher insulin secretion than those in static culture with a maximum 60-fold difference.

## Discussion

The overarching conclusion from the present study is that feline ASCs isolated from reproductive adipose tissue have endodermal transdifferentiation capabilities, and expression of some transcription factors, primarily those associated with progenitor cell maturation, is distinct between male and female donors over the course of differentiation. The resulting cell clusters secrete insulin in response to glucose stimulation in a dose-dependent manner, and cluster intracellular insulin and sensitivity to glucose levels is enhanced by the presence of theophylline or exposure to mechanical forces in the early stages of differentiation. Gene and protein expression as well as immunohistochemistry suggests transdifferentiation into a mixture of polyhormonal pancreatic progenitor cells and monohormonal functional pancreatic cells. This was further supported by cell micro- and ultrastructure consistent with pancreatic endocrine cells and the presence of functional metabolic regulatory proteins based on proteomic analysis. Potential insulinoma formation is unlikely based on the insulin secretion behavior and gene expression profile. Mechanical agitation during the first two stages of differentiation appears to promote maturation of insulin-secreting cells. Together these results confirm the ability to generate glucose-responsive cell clusters of pancreatic endocrine cells from feline reproductive adipose tissue adult ASCs. The results of this work may provide a platform for generation of viable, implantable organoids to restore native glucose regulation in cats and a multitude of species.

Heterogeneous primary cell isolates containing variable immunophenotypes and maturity levels were used to test the differentiation protocol. It was sufficiently robust for consistent cell differentiation and behavior despite likely differences among isolates (Ong et al., 2021). Cell characterization was limited to selection by plastic adherence and confirmation of adipocytic and osteoblastic mesodermal plasticity, as detailed analyses of the cell isolates are described in a previous publication utilizing the isolation method used in this study (Zhang et al., 2014). Based on that work, the isolates were expected to contain cells that were positive for the mesodermal stemness markers CD9, CD29, CD44, CD90, and CD105. It is possible that selection of a specific cell immunophenotype like CD177+ cells may increase the rate and efficiency of differentiation, though the size of initial cell isolates would likely be decreased (Mahaddalkar et al., 2020).

The three-step induction protocol developed in this study is distinct from previously published protocols and customized for feline reproductive adipose tissue ASC plasticity and behavior (Fargason et al., 2018). Specifically, N-2 and B-27 supplements were added to all 3 induction media (Dave et al., 2013) to enhance proliferation and limit apoptosis without the use of FBS, long known to have variable composition and stimulate immunity (McIntosh et al., 2009). Ultralow attachment culture plates used during stage 1 are an established mechanism to form spheroids from monolayer cell cultures (Chandra et al., 2011). Cells were cultured in standard cultureware at stage 2 to select for those that assumed an endodermal versus mesodermal lineage, the latter of which adhered to the plastic (Dominici et al., 2006; Okura et al., 2009). The use of laminin-coated plates for induction stage 3 was to promote spheroid survival and insulin secretion as reported for human and murine pancreatic islet isolates (Sigmundsson et al., 2018). Betacellulin was added to stage 3 medium since it promotes human embryonic stem cell differentiation into pancreatic endocrine cells (Cho et al., 2008; Pokrywczynska et al., 2013). Finally, the time required for each induction stage was based on the cell behaviors through numerous iterations. Taken together, the differentiation protocol described here represents a custom mixture of components for successful generation of pancreatic endocrine cells from feline reproductive organ adipose tissue ASCs.

Upregulation of *β* cell transcription factors and GCG, insulin, and STS confirmed transdifferentiation and maturation into distinct cell phenotypes, respectively (Chandra et al., 2009; Chandra et al., 2011; Xin et al., 2016). Specifically, increased expression of transcription factors associated with *β* cell lineage, Nkx6.1, and Pax6 (Pagliuca and Melton, 2013; Swisa et al., 2017; Aigha and Abdelalim, 2020), and function, Isl-1 and Glut-2 (Thorens, 2015; Wang et al., 2016), with increasing differentiation stage supports the presence and maturation of *β* cells. The transcription factor NKX6.1 is uniquely expressed in *β* cells of the adult pancreas (Gefen-Halevi et al., 2010). In contrast, the transcription factor Neurod1 interacts with others during pancreatic development to regulate progenitor cell lineage specificity among the multiple pancreatic endocrine cells, including *α*, *β*, and *δ* cells, among others (Naya et al., 1997; Chakrabarti and Mirmira, 2003; Mastracci et al., 2013). The effect of sex on MSC properties including, gene expression, surface marker expression, differentiation potential, and immunomodulation is well documented and distinct among MSCs of various tissue deposit origins (Corsi et al., 2007; Siegel et al., 2013; Xu et al., 2017; Bianconi et al., 2020; Knewtson et al., 2022). Nestin is a neural stem cell marker that is present within developing and adult pancreatic islets, and nestin expressing cells have the capacity to differentiate into pancreatic endocrine phenotypes (Zulewski et al., 2001; Eberhardt et al., 2006). The maximal expression of nestin has been reported to concur with a shift from proliferation to differentiation in undifferentiated cells (Kim et al., 2010), confirming a potential dual role as an intermediate regulator of both stemness and differentiation into insulin-secreting cells. Significantly higher expression of the gene in cells from female compared to male donors at stages 1 and 2 of differentiation may indicate an earlier transition to differentiation. This is consistent with the increased expression by male donor cells at stage 3 of differentiation when expression began to decline in cells from female donors. The earlier increase of Neurod1 expression in cells from female donors that was significantly higher than in male donor cells further substantiates this finding. Notably, by the conclusion of stage 3 of differentiation, the differences in gene expression between donor sexes were no longer evident.

As alluded to above, the cell clusters were likely a combination of poly- and monohormonal pancreatic endocrine cells at the completion of the third culture stage. The presence of polyhormonal cells is not unexpected given that pancreatic embryogenesis involves differentiation of common progenitors into distinct lineages (Pagliuca and Melton, 2013), and resident pancreatic adult progenitor cells have the capacity to commit to distinct cell types as needed for organ repair and homeostasis (Romer and Sussel, 2015). Consistent with the changes in gene expression described above, cell micro- and ultrastructure confirmed cell maturation as well. Mature *β* cells were suggested by positive staining of insulin zinc and confirmed by bright field immunohistochemistry and immunoelectron microscopy, the latter of which showed insulin localized within intracellular vacuoles (D'Amour et al., 2006). The presence of proteinaceous granules on the surface of cells cultured in differentiation medium could represent *α* or *β* cell secretory granules as could the appearance of secretory vacuoles, a hallmark of pancreatic endocrine cells, evident with electron microscopy (Gao et al., 2008; Xie et al., 2009; Pfeifer et al., 2015), though vacuole contents were lost during processing in the latter. Single cell labeling was not clear with immunohistochemical localization of insulin, glucagon, and somatostatin, but distinct cell phenotypes were confirmed with Mallory-Heidenhan Azan-Gomori staining (Baskin, 2015) and suggested by the proteomic profile. Together, these results support the islet like composition of the cell clusters produced for this investigation. However, preclinical testing in an established diabetes model is necessary to confirm the ability of the clusters to regulate systemic glucose levels as well as the duration of cluster activity, which will vary with implantation location (Kottaisamy et al., 2021).

The insulin secretion by functional *β* cells in response to glucose challenges was consistent with the expected response of increasing insulin secretion with glucose concentration (Gao et al., 2008). Lack of both insulin secretion at the magnitude of functional islets and a biphasic pattern of insulin release is a standard finding in most reports of differentiated insulin-producing cells from various progenitor cells, including induced pluripotent stem cells (Pagliuca et al., 2014). This has recently been reported to be a result of failure of reduced anaplerotic cycling in the mitochondria (Davis et al., 2020). Failure of clusters to return to low levels of insulin secretion at low glucose concentrations may also indicate cell senescence characteristic of *in vivo β* cell failure (Hasnain et al., 2016). Exposure to theophylline enhanced insulin secretion and content in clusters generated under static culture, though only at the higher glucose concentrations, likely due to lower sensitivity of fewer and less mature *β* cells. Enhanced insulin secretion by cells grown in dynamic versus status culture suggests a greater number or greater maturity of *β* cells. The relative lack of glucagon staining with dynamic culture may indicate a greater number of monoclonal *β* cells and a cell population more consistent with the native ratio of *β* to *α* cells in the feline pancreas, approximately 8:1 (Zini et al., 2016). The limitations of light microscope imaging may have precluded identification of a low number of glucagon expressing cells within clusters.

Shear stress from blood flow plays an essential role in regulating *β* cell polarity required for specialization during pancreatic development (Lodh et al., 2014). Dynamic medium flow through bioreactors during induced pluripotent stem cells differentiation to IPCs reportedly increases cluster *β* cell gene expression (*NKX6.1, INS*) and glucose-stimulated insulin secretion over static culture (Tao et al., 2019). An additional consideration is cluster size, and the dynamic culture employed in this study utilizing 2.5 × 10^4^ cells/cm^3^ resulted in relatively homogeneous clusters close in size to the native feline islet (Zini et al., 2016; Brandão et al., 2018). Homogenous cluster size may contribute to a predictable cell composition, and reduced cell viability in larger clusters likely impacted function. A previous investigation confirmed that reducing the size clusters to 172 from 364 μm improved insulin secretion by β-like cells derived from a human embryonic cell line (Velazco-Cruz et al., 2019). It is possible that addition of theophylline to the culture medium in combination with dynamic culture may decrease culture times and enhance progenitor cell differentiation into distinct endocrine cells of functional organoids (Gabr et al., 2014).

While not definitive, the insulin secretion response and gene expression profile of the clusters produced in this study is not consistent with that of insulinomas. As detailed above, differentiated clusters exhibited some level of regulated insulin secretion while insulinomas tend to have exceedingly high, unregulated insulin secretion in response to all glucose concentrations, high or low (Henquin et al., 2015). The increase after stage 2 in RAB3A gene expression, a small GTPase associated with vesicle trafficking and exocytosis, including insulin secretion, coincides with the transcription factor transitions associated with cell differentiation described above (Takai et al., 1996; Sun et al., 2001; Yaekura et al., 2003; Kasai et al., 2005; Vieira, 2018). Notably, RAB3 plays a role in regulated versus constitutive secretion, and RAB isoforms are expressed almost exclusively in neurons and secretory cells (Raffaniello, 2021). In contrast to normal feline *β* cells, insulinoma cells express the HK1 gene and do not show glucagon protein expression, similar to lacking glucagon protein expression within *Men1*-ablated murine insulinomas (Jackson et al., 2009; Li et al., 2015). The HK1 expression in clusters cultured in stromal medium that was about double that of those in differentiation medium as well as the presence of glucagon in clusters substantiates, at minimum, a lower potential for insulinoma formation. Members of the AKT family are part of signaling cascades that positively regulate *β* cell mass and insulin secretion; however, AKT1 overexpression is associated with glucose tolerance and insulinoma formation (Alliouachene et al., 2008). The initial increase in AKT1 expression that decreased with differentiation is consistent with *β* cell maturation, and the return to basal levels inconsistent with insulinoma. Finally, islet-like clusters produced from human MSCs that formed tumors in mice 40–45 days post transplantation lacked expression of key pancreatic transcription factors, Nkx6.1 and Pax6, which where both highly upregulated in the differentiated clusters of this study (Tang et al., 2012).

These study results confirm mesenchymal-endodermal transdifferentiation by male and female feline reproductive adipose tissue ASCs and the potential to generate functional pancreatic organoids with diverse cell populations of native pancreatic islets. Mechanisms to scale up cluster production and reduce time for differentiation will likely be necessary for clinical implementation, and addition of a methylxanthine like theophylline to differentiation media combined with dynamic culture may improve efficiency. *In vivo* cluster behavior must be tested to confirm the magnitude and duration of insulin secretion as well as long term maintenance of cell lineages that do not contribute to tumorigenesis. Despite these acknowledged limitations, the findings of these investigations provide a clear mechanism to advance the science of *de novo* tissue generation from adult MSCs to address problems with high clinical relevance.

## Data Availability

The original contributions presented in the study are included in the article/supplementary materials. Further inquiries can be directed to the corresponding author.
